# The new Zero-P implant can effectively reduce the risk of postoperative dysphagia and complications compared with the traditional anterior cage and plate: a systematic review and meta-analysis

**DOI:** 10.1186/s12891-016-1274-6

**Published:** 2016-10-18

**Authors:** Mengchen Yin, Junming Ma, Quan Huang, Ye Xia, Qixing Shen, Chenglong Zhao, Jun Tao, Ni Chen, Zhingxing Yu, Jie Ye, Wen Mo, Jianru Xiao

**Affiliations:** 1 Department of Orthopaedics, LongHua hospital, Shanghai university of Traditional Chinese Medicine, Shanghai, People’s Republic of China; 2 Department of Bone Tumor Surgery, Changzheng Hospital, Second Military Medical University, Shanghai, People’s Republic of China

**Keywords:** Systematic review, Cervical spine, Cervical spondylosis, Cage, Zero-P plate

## Abstract

**Background:**

The low-profile angle-stable spacer Zero-P is a new kind of cervical fusion system that is claimed to limit the potential drawbacks and complications. The purpose of this meta-analysis was to compare the clinical and radiological results of the new Zero-P implant with those of the traditional anterior cage and plate in the treatment of symptomatic cervical spondylosis, and provides clinicians with evidence on which to base their clinical decision making.

**Methods:**

The following electronic databases were searched: PMedline, PubMed, EMBASE, the Cochrane Central Register of Controlled Trials, Evidence Based Medicine Reviews, VIP, and CNKI. Conference posters and abstracts were also electronically searched. The efficacy was evaluated in intraoperative time, intraoperative blood loss, fusion rate and dysphagia.

**Results:**

For intraoperative time and intraoperative blood loss, the meta-analysis revealed that the Zero-P surgical technique is not superior to the cage and plate technique . For fusion rate, the two techniques both had good bone fusion, however, this difference is not statistically significant. For decrease of JOA and dysphagia, the pooled data showed that the Zero-P surgical technique is superior to the cage and plate technique.

**Conclusions:**

Zero-P interbody fusion can attain good clinical efficacy and a satisfactory fusion rate in the treatment of symptomatic cervical spondylosis. It also can effectively reduce the risk of postoperative dysphagia and its complications. However, owing to the lack of long-term follow-up, its long-term efficacy remains unknown.

## Background

Cervical spondylosis is a frequent cause of radicular arm pain, and is a leading cause of spinal cord dysfunction in adults. Surgical treatment is indicated when conservative therapy fails or when the symptoms worsen [1, 2]. In 1958, Cloward, Smith, and Robinson first reported that anterior cervical operation is a safe and effective method for the treatment of degenerative cervical spondylosis. Anterior cervical discectomy and fusion (ACDF) is still performed in most cases and is the golden standard for the treatment of cervical spondylosis, regardless of whether a single segment or multiple segments are involved [3–5]. At present, cages are widely used for cervical fusion clinically. These techniques have their own benefits as well as potential drawbacks and adverse effects. The most often mentioned shortcomings of these techniques are sacrifices of the original activity of the segment and changes in stress distribution of the adjacent segment [6, 7]. These will accelerate disc degeneration and cause many complications, such as postoperative dysphagia for the anterior plate constructs [8–10]. Many studies have reported that an anterior plate with a lower, smoother profile may reduce the incidence of dysphagia after ACDF [11, 12].

Low-profile angle-stable spacer Zero-P, which was approved by the United States Food and Drug Administration in 2008, is a new kind of cervical fusion system that has been declared to limit the potential risk of these drawbacks and complications. The design of Zero-P is based on the polyetheretherketone (PEEK) intervertebral fusion Syncage –C, where the small titanium plate is integrally inserted into the intervertebral disc, which is then secured to the upper and lower vertebral body by the insertion of screws. The advantages of using intervertebral fusion cage and plate can also be attained via Zero-P. In particular, it can increase the immediate stability of treated segment even in the absence of an anterior implant, and decrease the incidence of postoperative dysphagia and adjacent segments degeneration. There is still no comprehensive review regarding the comparative analysis on outcomes between these two cervical spine procedures. Consequently, this article is the first meta-analysis based on all literatures to compare clinical and radiological results of the new Zero-P implant with those using traditional anterior cage and plate in the treatment of symptomatic cervical spondylosis, and to provide clinicians with an evidence base for their clinical decision making.

## Methods

### Search strategy

The research group followed the recommendations of Preferred Reporting Items for Systematic Reviews and Meta-Analyses (PRISMA) for this meta-analysis [13]. The following electronic databases were searched from their inception dates through August 2013: Medline, PubMed, EMBASE, the Cochrane Central Register of Controlled Trials, Evidence Based Medicine Reviews, VIP, and CNKI. The study used Boolean logic with search terms including “Zero-P”, “cervical spondylosis,” and “cervical fusion.” The references for all located articles, including other systematic reviews, were searched manually for additional relevant articles.

### Inclusion criteria

#### Types of studies

The research group included all studies comparing the new Zero-P implant with the traditional anterior cage and plate in the treatment of symptomatic cervical spondylosis. Studies that provided no information about complications and had no specific data on the clinical effect were excluded. All basic research reports (biomechanics and basic science studies) were also excluded.

### Types of participants

The research group excluded trials in which a specific cervical disease could be identified, such as fracture, trauma, developmental cervical stenosis, ossification of the posterior longitudinal ligament, and a previous cervical spinal surgery.

### Types of outcomes

#### Surgical details

Intraoperative time and intraoperative blood loss are important objective bases for the evaluation of operation situation. Therefore, they can have objective through comparison the evaluation of operation trauma.

### Radiographic outcomes

The procedure of using a traditional cage combined with a plate has been recognized to have a good bony fusion rate. This review aimed to determine the outcome of the new Zero-P device through the comparison of the fusion rates of the operated segments at the last follow-up.

### Clinical outcome

#### Japanese Orthopaedic Association (JOA) score

The review was more pertinent than other studies with regards to comparing the two operative procedures for treating cervical degenerative disease. Therefore, research group set the decrease of JOA score as the primary outcome, in the evaluation of clinical outcome, which was assessed at the last follow-up after the intervention.

### Dysphagia and its complications

Dysphagia and its related complications are common after anterior cervical fusion surgery, and its incidence varies in the literature. Therefore, this review also focuses on evaluating postoperative dysphagia and its complications as adverse events in order to compare the efficacy of the two procedures.

### Data extraction and validity assessment

All the study characteristics and outcome data were extracted from all the included studies independently. The research group extracted the details of trials, and if certain elements were missing, respective study authors were contacted to obtain the relevant missing data. Any differences in opinion about eligibility were resolved by consensus.

### Assessment of methodological quality and heterogeneity

Two independent reviewers assessed the methodological quality by using the Newcastle-Ottawa Scale, while modifying it to match the needs of this study [14, 15]. The quality was evaluated by examining three items: selection, comparability, and exposure, with higher scores representing studies of higher quality. The quality of each study was graded as either level 1 (0–5) or level 2 (6–9) [16]. This review also assessed the clinical heterogeneity to evaluate whether the trials were similar enough to pool data.

### Assessment of risk bias

The risks of bias of all included trials were also independently assessed by the other two reviewers according to the criteria of the Cochrane Handbook for Systematic Reviews.

### Data analysis

In the review, for categorical data, dysphagia and its complications were dichotomized into two categories. The effect size for a reported decrease of JOA score was defined as a pooled estimate of the weighted mean difference (WMD) in the change.

The heterogeneity of all the studies was determined by I^2^ statistics. If the I^2^ value was >50 %, the study marked it as a considerable level of heterogeneity; otherwise, it was considered to be of good homogeneity. The research group also assessed clinical heterogeneity. Statistically and clinically homogeneous studies were pooled using a fixed-effects model; otherwise, a random-effects model was used when the heterogeneity was significant.

## Results

### Study description and risk bias

**Fig. 1:**
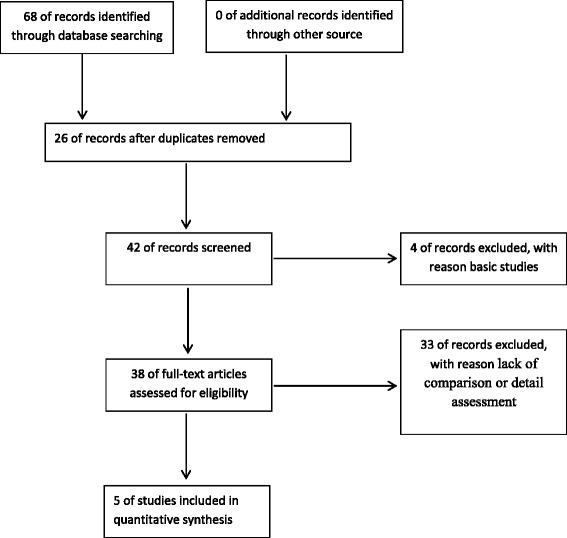
Select process

Finally, five studies were incorporated into the systematic review, with a total enrolment of 472 patients and available follow-up data [17–21]. Figure 1 shows the selection process. Four clinical trials are retrospective studies. Only one trial is a prospective study. Table 1 summarizes the demographic data from these studies, and Table 2 presents the assessment of methodological quality. None studies demonstrated randomization and sufficient allocation concealment. None described the blinding of outcome assessment, participants and personnel. All studies retained complete outcome data and avoided selective reporting. As a result, the overall quality of the included studies demonstrated a high risk of bias.

**Table 1:** Demographic data

	Design	Number		Age		Segment		Follow-up (year)
		Z	c + p	Z	c + p	Z	c + p	
Wang 2013	R	22	24	50.86 ± 8.79	53.33 ± 8.98	44	48	2
Petr 2013	P	44	33	50.2 ± 10.3	51.8 ± 10.9	55	41	2
Miao 2013	R	39	50	50.3 ± 25.9	52.6 ± 23.7	71	97	2
Hofstetter 2013	R	35	35	56.8 ± 1.6	51.5 ± 2.0	53	54	2
QI 2013	R	83	107	43.6 ± 26.7	44.9 ± 27.4	175	225	1.5

*Abbreviations*: *N* number of patient, *Z* Zero-P group, *c + p* cage and plate group, *P* Prospective Study, *R* Retrospective Study

**Table 2:** Assessment of methodological quality

	selection			comparability		exposure		scores
	1	2	3	4	5	6	7	
Wang 2013		*	*	*	*		*	*****
Petr 2013	*	*	*	**	**	*	*	*********
Miao 2013		*	*	*	*	*	*	******
Hofstetter 2013	*	*	*	**	*	*	*	********
QI 2013	*	*	*	*	*	*	*	*******

### Surgical details

#### Intraoperative time

**Fig. 2:**
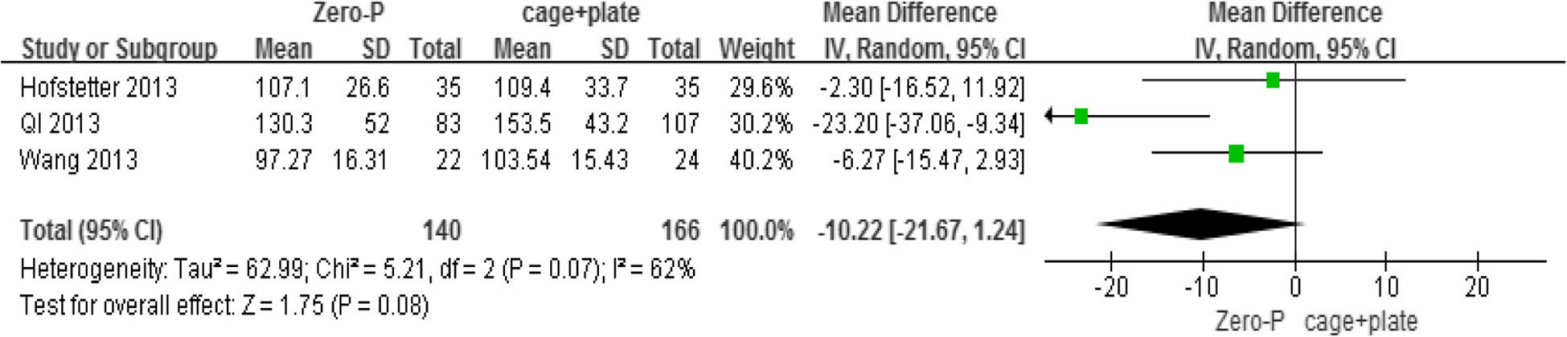
Zero-P versus cage + plate on intraoperative time

3 studies were selected for the criterion on intraoperative time (minutes). The pooled data showed that patients submitted to the Zero-P technique did not have a significantly shorter intraoperative time than those who underwent the cage and plate technique of cervical fusion, in the random-effects model (3 trials, *n* = 306, pooled WMD = −10.22, 95 % CI = −21.67 to 1.24, *P* = 0.08) (Fig. 2).

### Intraoperative blood loss

**Fig. 3:**
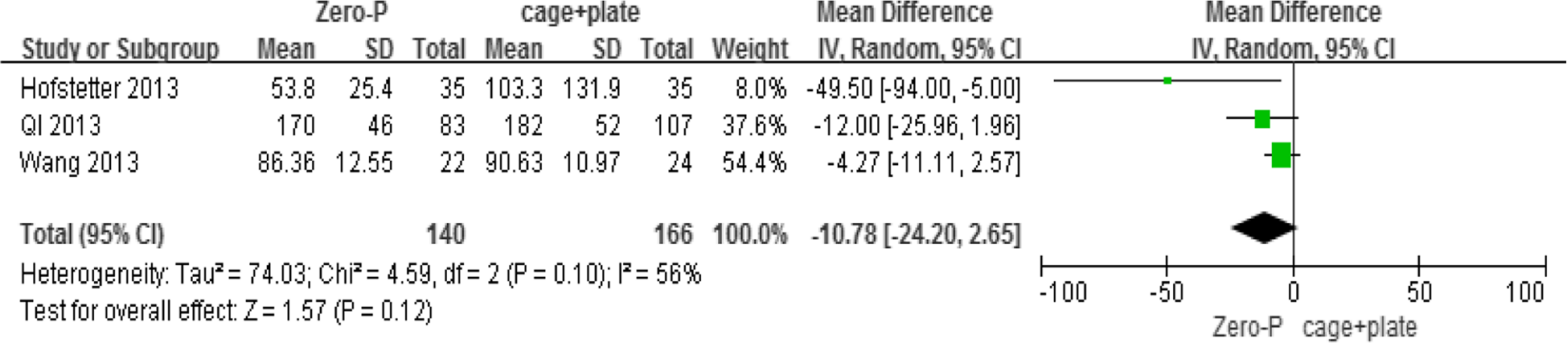
Zero-P versus cage + plate on intraoperative blood loss

For the outcome measure on Intraoperative blood loss (ml), three studies were selected. The meta-analysis revealed that the Zero-P surgical technique is not superior to the cage and plate technique for this outcome measure in the fixed-effects model (3 trials, *n* = 306, pooled WMD = −10.78, 95 % CI = −24.20 to 2.65, *P* = 0.12) (Fig. 3).

### Radiographic outcome

#### Fusion rate

The results of the three studies showed that all surgical levels in both groups had good bone fusion. In one study, all patients who were submitted to the interbody technique presented better results for this outcome. All surgical levels showed good fusion after the operation in three trials. One trial reported that 97.1 % in the Zero-P group and 100 % in the cage + plate group had good bone fusion; however, this difference is not statistically significant.

### Clinical outcome and complications

#### Decrease of JOA score

**Fig. 4:**
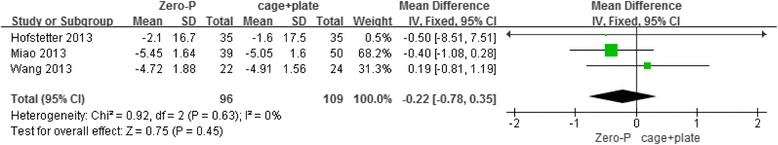
Zero-P versus cage + plate on intraoperative decrease of JOA score

Three studies used the decrease in JOA score to evaluate the clinical outcome. The pooled data showed that in terms of the decrease in JOA score, Zero-P was not more effective than ACDF in the fixed-effects model (3 trials, *n* = 205, pooled WMD = 0.22, 95 % CI = −0.78 to 0.35, *P* = 0.75) (Fig. 4).

### Dysphagia (early postoperative period)

**Fig. 5:**
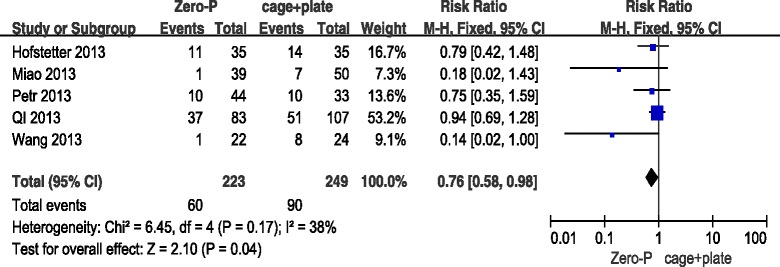
Zero-P versus cage + plate on dysphagia early postoperative

All five studies reported dysphagia in the early postoperative follow-up for both the Zero-P and the cage + plate group. The pooled data showed that the Zero-P group had a lower incidence of dysphagia and complication compared with the ACDF group in the fixed-effects model (5 trials, *n* = 472, pooled RR = 0.76, 95 % CI = 0.58 to 0.98, Z = 2.10, *P* = 0.04) (Fig. 5).

### Dysphagia (at last follow-up)

**Fig. 6:**
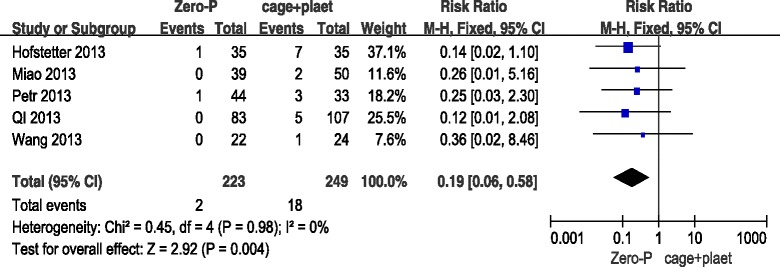
Zero-P versus cage + plate on dysphagia at last follow-up

All five studies also described difficulty in swallowing at the last follow-up for both the Zero-P and the cage + plate group. The difference between the two groups was statistically significant. The incidence of dysphagia was lower at the last follow-up in the Zero-P group than in the ACDF group in the fixed-effects model (5 trials, *n* = 472, pooled RR = 0.19, 95 % CI = 0.06 to 0.58, *Z* = 2.92, *P* = 0.004) (Fig. 6).

## Discussion

Anterior cervical decompression by discectomy followed by fusion is a widely accepted and safe surgical procedure for the treatment of degenerative cervical spine disease [22]. The primary aim of this technique is to decompress the spinal cord and the affected nerve roots while restoring cervical alignment.

Seventy to 80 % of cases that were treated with complete discectomy alone led to spontaneous bony fusion; therefore, anterior cervical discectomy with or without interbody fusion is a widely accepted technique [23]. However, this technique is criticized because it does not maintain the cervical curvature, prevent instability and osteophyte formation, and it does not preserve the vertebral disc height. Consequently, intervertebral fusion is widely recommended [24]. Initially, the iliac was often used as a substrate to achieve bone fusion. However, it may bring a considerable risk of donor site morbidity in the cervical anterior fusion technique [25, 26]. To prevent these adverse events, many kinds of bone graft substitutes and cages have been investigated.

The stand-alone technique in anterior cervical interbody fusion is done by cage insertion without any additional support. It is a widely accepted and a proven method because not only can stand-alone cage insertion help restore foraminal height, it also provides immediate load-bearing support to the anterior column and facilitates arthrodesis [27, 28]. However, there is still considerable controversy about the stand-alone cage technique because of complications such as anterior cage migration, lower immediate stability with the cage, segmental kyphosis, and cage subsidence [6, 7].

Maintenance of the cervical curvature and disc height, prevention of cage subsidence, and conferring greater stability to the operated segment are often mentioned as reasons for the implantation of an anterior plating system. In addition, it can prevent the development of kyphosis and increase the rate of bone fusion [10, 29, 30]. Thus, the fusion procedure with a cage and a plate seems to be the golden standard in the treatment of patients with symptomatic cervical spondylosis. Nevertheless, plate migration, acceleration of disease of the adjacent segment, and dysphagia are the most frequently mentioned drawbacks associated with the anterior cage and plate technique [31, 32].

The intervertebral fusion device named Zero-P is a new kind of cervical fusion system that can be independently applied to single-segment or a multi-segment anterior cervical spondylosis [8]. This device has the benefits of both the cage and the anterior plate. A Zero-P fusion implant in the intervertebral space after decompression will not be prominent in the vertebral column. Owing to its design, Zero-P can significantly limit the potential risks of postoperative dysphagia and degeneration of adjacent segments after the internal fixation in anterior cervical fusion surgery. Furthermore, and in particular, it can increase the immediate stability of the treated segments [33].

The results of present review shows strong statistical evidence for the clinical efficacy of the two anterior cage systems in the treatment of symptomatic cervical spondylosis. It is important to emphasize that the quality of the evidence is relatively low due to the lack of randomized clinical trials (RCTs); moreover, the included observational studies may have selection bias and most studies had a small sample size, leading to a lack of statistical power [34].

In this review, it studied the relevant clinical outcomes, assessed the clinical outcome, and surgically related complications. This study confirms that the data are comprehensive enough to explore the clinical difference.

Postoperative dysphagia and its related complications are common after anterior cervical surgery. Its incidence rate reported in the literature varies from 1 to 70 % [35–43]. The associated significant findings indicate that the risk factors for postoperative dysphagia include the age of patients, characteristics of internal fixation and many others [44–47]. Studies have shown that the thickness of the anterior plate used in anterior cervical discectomy and fusion surgery is positively correlated to the incidence rate of postoperative dysphagia and its complications [33]. Therefore, reducing the thickness of the anterior plate could considerably reduce the rate of the incidence of postoperative dysphagia and its complications. The study verified in this review shows that the incidence rate of postoperative dysphagia in the Zero-P group was significantly lower than that in the cage + plate group during first 3 months of postoperative follow-up and at the last follow-up. This might be because the design of Zero-P allowed avoiding the stimulus production of the anterior cervical plate to the esophagus and surrounding soft tissues.

Studies have shown that the Zero-P implant could demonstrate similar biomechanical properties as the traditional anterior cervical cage and plate, such as allowing good activity and stability of the surgical segments [48]. The group of Scholz et al. also selected 38 patients with cervical spondylosis who underwent Zero-P cervical interbody fusion surgery for an average of 8 months follow-up. The follow-up results showed that all patients obtained satisfactory bone fusion and functional recovery [49]. In this review, the results suggest that the new Zero-P internal fixation system could lead to a good fusion rate similar to that with the traditional cervical fusion surgery with a cage and anterior plate. For intraoperative time and intraoperative blood loss, it was difficult to conclude which is the better technique in terms of operation trauma. Various ages of patients, degree of degeneration or skills of surgical team may be the reasons for heterogeneity in intraoperative time and blood loss.

Degeneration of adjacent segments is another common complication in anterior cervical fusion surgery, especially in multilevel procedures and with the use of an oversized plate. The result show that there was no statistical difference between the two surgical procedures. However, further analysis was not possible because of the inability to perform data merging. In addition, the long-term effects of adjacent segment degeneration are unknown for the two procedures.

In this review, satisfactory fusion was achieved by both forms of surgical treatment, with no significant difference between the two groups. Thus, Zero-P produces as good a rate of fusion and biomechanical stability as does a plate and cage construct, and both procedures corrected cervical kyphosis and improved cervical alignment.

However, Zero-P also has some disadvantages. For example, the lower screws of C3/4 and the upper screws of the C6/7 implant are relatively difficult to insert at an optimal angle, even if the patient does not have a short muscular neck. The increased retraction and a need for a wider skin incision also need to be considered [20].

Furthermore, many relevant issues remain unresolved: for example, whether the difference in the incidence rates of postoperative complications between the two groups are related to the surgical segments, the amount of bleeding, or other factors; whether sinking of the cage will occur; the long-term stability of the treated segment; and whether kyphosis and other complications will occur. These issues are expected to be explored and analyzed in future clinical studies.

This study has several notable strengths. First, this study confirm that this review is the first to compare the clinical effects between the new Zero-P implant with the traditional anterior cage and plate in the treatment of symptomatic cervical spondylosis. Second, this study aimed to investigate the difference of the clinical effects of the two cervical anterior fusion surgeries, and judged whether the difference is clinically important. With this objective, this study conducted a systematic literature search to guarantee the comprehensiveness of the included trials. The research group believe that this is the most comprehensive and largest review thus far considering clinical outcome and surgically related complications. The included trials studied a large number of patients, and this review has adequate statistical power to analyze and explore the difference in clinical effects, despite the exclusion of numerous clinical trials because of having a low level of evidence (no control group).

However, results also have some limitations. First, this review only included five clinical trials with a relatively small sample size. Moreover, the five trials included were observational comparative studies but only one study was prospective. The prospective clinical trial did not undergo the appropriate randomization and allocation concealment. Therefore, in future clinical work, high-quality, large-sample, and multi-centered randomized and controlled clinical trials should be conducted as far as possible to provide doctors with the best evidence-based information for the treatment of cervical spondylosis. To improve the trial design quality and the level of performance, future trials should follow the guidelines for reporting clinical trials such as the CONSORT statement [50, 51]. In addition, the follow-up period of all the included trials were relatively short, with the longest being 2 years; thus, the long-term clinical effect remains unknown. In addition, relevant related information about adjacent segment degeneration was unreported. Therefore, our research failed to draw a conclusion about these issues.

## Conclusions

Both the new Zero-P interbody fusion and the traditional anterior cage and plate construct can attain good clinical efficacy and satisfactory fusion rate in the treatment of symptomatic cervical spondylosis. Zero-P can effectively reduce the risk of postoperative dysphagia and its complications, while demonstrating good clinical efficacy. However, owing to the lack of long-term follow-up, its long-term efficacy remains unknown. Large sample sizes and high-quality multicenter clinical trials are needed for a deeper analysis of Zero-P interbody fusion.

## Abbreviations

c + p: cage and plate group
N: Number of patient
P: Prospective study
R: Retrospective study
Z: Zero-P group
